# Surgical Outcome of Brunner's Gland Hamartoma: A Single-Centre Experience

**DOI:** 10.1055/s-0041-1741510

**Published:** 2022-01-17

**Authors:** Arkadeep Dhali, Sukanta Ray, Tuhin S. Mandal, Somak Das, Ranajoy Ghosh, Sujan Khamrui, Gopal K. Dhali, Avik Sarkar

**Affiliations:** 1 Department of Gastrointestinal Surgery, Institute of Postgraduate Medical Education and Research, Kolkata, West Bengal, India; 2 Department of Gastrointestinal Pathology, Institute of Postgraduate Medical Education and Research, Kolkata, West Bengal, India; 3 Department of Gastroenterology, Institute of Postgraduate Medical Education and Research, Kolkata, West Bengal, India; 4 Department of Gastrointestinal Radiology, Institute of Postgraduate Medical Education and Research, Kolkata, West Bengal, India

**Keywords:** Brunner's gland hamartoma, duodenal polyp, surgery, outcome

## Abstract

**Introduction** Brunner's gland hamartomas (BGH) are rare benign lesions with an incidence of <0.01%, accounting for 5 to 10% of all benign tumors of the duodenum. It requires expeditious management by a multidisciplinary team. The aim of the study is to report our experience with surgery for BGH.

**Methodology** Data of all patients who underwent surgical intervention for duodenal polypoidal mass between August 2007 and March 2020 were retrieved from our prospectively maintained gastrointestinal (GI) surgery database. All patients whose histopathology report of the resected specimen confirmed BGH ( *n* = 9) were included in the present study. Other pathological diagnosis like duodenal lipoma ( *n* = 2), ganglioneuroma ( *n* = 1), adenoma ( *n* = 10), and adenocarcinoma ( *n* = 4) were excluded.

**Results** Nine patients had confirmatory histopathological diagnosis of BGH and met our inclusion criteria. Three (33.3%) of them were men with a median age of 45 (range: 24–61) years. The median interval between onset of symptoms and diagnosis of duodenal polyp was 14 (range: 4–180) days. Five patients (55.5%) presented with upper GI hemorrhage. Three (33.3%) patients presented with abdominal pain, and one (11.1%) patient presented with episodes of bilious vomiting. Diagnostic endoscopy could detect the lesion in all (100%) patients. Contrast-enhanced computed tomography detected duodenal polypoidal lesion in five (55.5%) patients. The mean size of tumor was 4.78 ± 1.36 cm. These lesions were symptomatic in all the patients and warranted intervention. In view of failed endoscopic intervention ( *n* = 7, 77.7%), or extramural extension of the tumor ( *n* = 2, 22.2%), surgical intervention was considered. Most commonly performed operation was duodenal polypectomy ( *n* = 6, 66.6%). Three postoperative complications developed in two (22.2%) patients. There was no surgery-related mortality. After a median follow-up of 60 (12 -78) months, no patient developed GI bleed or intestinal obstruction.

**Conclusion** In this study, the clinical profile of BGH was explored from the surgeon's point of view. Although endoscopic management is the first-line treatment, surgery plays an important role, particularly, if this fails or is not feasible. In experienced hand, surgery can be performed with acceptable perioperative morbidity and mortality and long-term satisfactory outcomes.

Brunner's gland hamartomas (BGH) are rare benign lesions with an incidence of <0.01%, accounting for less than 1% of all primary tumors of the small intestine. 1 They are commonly found in the first and second part of the duodenum. They may be diagnosed incidentally in asymptomatic cases or may cause upper gastrointestinal (GI) hemorrhage, iron deficiency anemia, or duodenal obstruction. Endoscopy has both diagnostic, as well as therapeutic implications. Less than 100 cases are reported in existing medical literature and the data relies on individual case reports or review articles. 1 Due to rarity of the disease and paucity of data, uniform management guidelines are nonexistent. Although surgical resection is reserved for cases where endoscopic snare dissection is either not possible or has failed. 2 Our center is one of the surgical gastroenterology referral centers where patients with failed endoscopic management are referred for surgical intervention. The aim of the study is to report our experience with surgery for BGH.

## Methodology

This is a retrospective observational study. Data of all patients who underwent surgical intervention for duodenal polypoidal mass between August 2007 and March 2020 were retrieved from our prospectively maintained GI surgery database. All patients whose histopathology report of the resected specimen confirmed BGH were included in the present study. Other pathological diagnosis like duodenal lipoma ( *n* = 2), ganglioneuroma ( *n* = 1), adenoma ( *n* = 10), and adenocarcinoma ( *n* = 4) were excluded.

This study was approved by the institutional ethics committee (Memo number: IPGME&R/RAC/266, dated September 10, 2021). Informed patient consent was waived by the ethics committee due to the retrospective nature of the study and also as the data were anonymized.

### Diagnosis

**Fig. 1:**
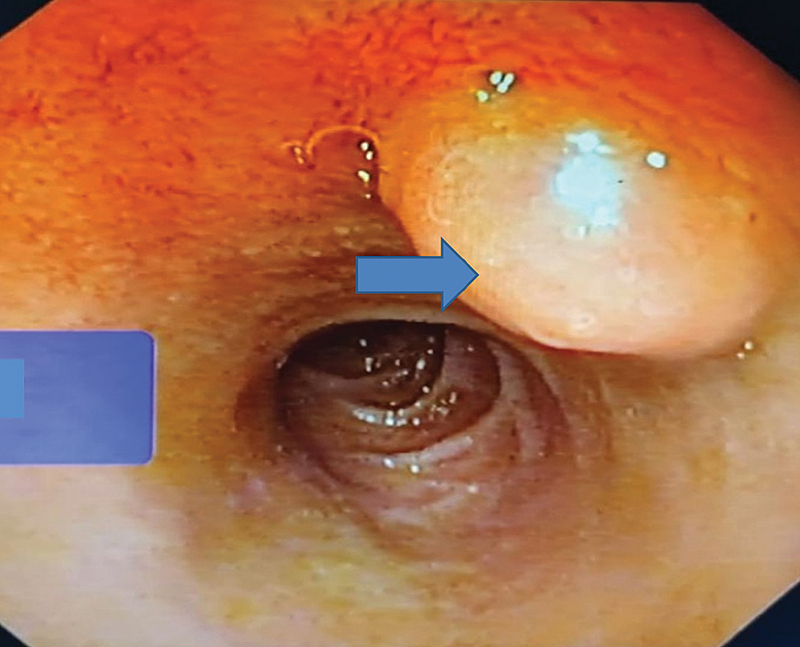
Esophagogastroduodenoscopy image showing a smooth sessile polypoidal swelling with a large base in the first part of the duodenum in the posterior wall of the duodenal bulb with no ulcer or bleeding (blue arrow).

**Fig. 2:**
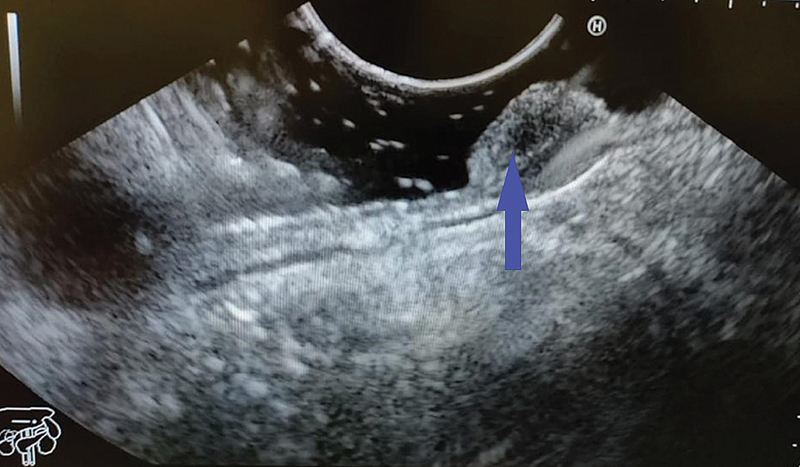
EUS showing a 2.2-cm hyperechoic lesion arising from the submucosal layer with no calcification, cystic change, or ductal structure. EUS, endoscopic ultrasound.

**Fig. 3:**
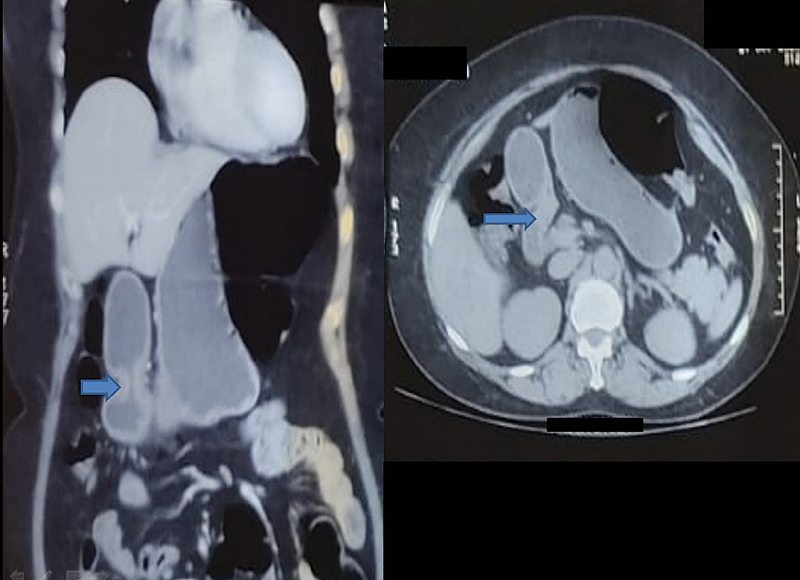
Contrast-enhanced computed tomography showing a homogeneously enhancing polypoidal mass arising from the posterior wall of duodenal bulb.

**Fig. 4:**
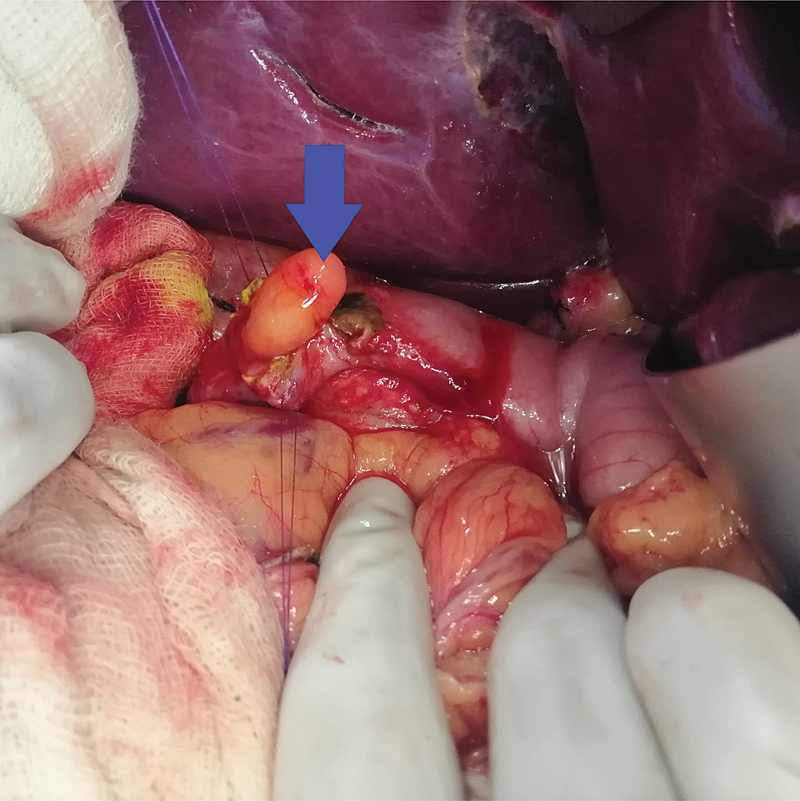
Operative photograph showing a polyp arising from the first part of the duodenum.

**Fig. 5:**
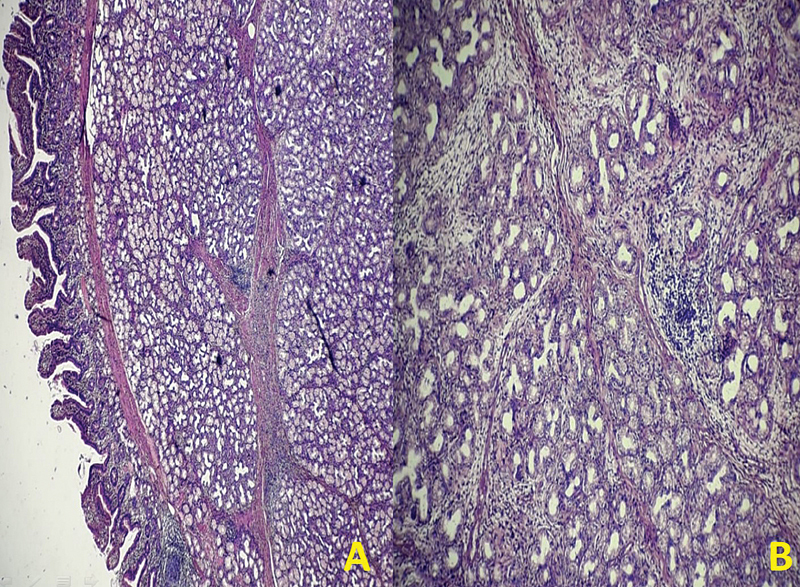
( **A and B** ) H&E image showing an admixture of fibrovascular tissue, adipose tissue, and hyperplastic Brunner's gland extending to lamina propria from submucosa suggestive of Brunner's gland hyperplasia (BGH). H&E, hematoxylin and eosin.

Clinical features of upper GI hemorrhage (hematemesis, melena) and obstructive symptoms (bilious vomiting and pain abdomen) warranted an endoscopic evaluation ( Fig. 1 ). Polypoidal lesions were visualized in first or second part of duodenum. Endoscopic ultrasound (EUS; Fig. 2 ) was used to characterize the nature (solid or cystic, size, extent, and vascularity), as well as the layer of origin (submucosa) of the lesion. To look for any extraluminal extension of the mass, cross-sectional imaging, like contrast-enhanced computed tomography ( Fig. 3 ), was performed. BGH is a histopathological diagnosis and hence surgically resected specimen ( Fig. 4 ) on pathological evaluation ( Fig. 5 ) confirmed the diagnosis.

### Treatment

The line of management was decided by a multidisciplinary team including gastroenterologists, radiologists, and GI surgeons. Patients presenting with hemodynamic instability were resuscitated with fluid replacement and blood transfusion. In cases of only intraluminal lesions, endoscopic snare polypectomy was attempted. In cases of large lesions which were obscuring lumen, sessile polyps, failed endoscopic removal, and lesions with extraluminal extension, surgery was performed. The type of surgery for BGH was determined by the site, size, and its relation to the adjacent organs like the pancreas, major duodenal papilla, and pylorus. In pedunculated polyp, duodenal polypectomy was performed. Large polyp with a sessile base required segmental or wedge resection of the duodenum with primary anastomosis. Duodenum was repaired with 4–0 polydioxanone (PDS) (single-layer interrupted stitches). When the large polyp was arising from the medial duodenal wall close to the major duodenal papilla, Whipple's operation was performed. Pyloroplasty was added when there was luminal narrowing after removal of the polyp in the first part of the duodenum close to the duodenal bulb.

### Definitions

Death during the hospital stay or within 90 days after the intervention was the definition of perioperative mortality utilized. Postoperative complications were graded using the Clavien–Dindo classification. 3 Pancreatic fistulae, postpancreatectomy hemorrhage, and delayed gastric emptying were defined and classified according to the criteria of International Study Group on Pancreatic Surgery (ISGPS). 4 5 6

### Statistical Analysis

Quantitative variables were expressed as mean ± standard deviation or median with range. Dichotomous variables were expressed as a percentage.

## Results

Nine patients had a confirmatory histopathological diagnosis of BGH and met our inclusion criteria.

### Demographic Details

Three (33.3%) of them were men with a median age of 45 (range: 24–61) years. Alcohol abuse was identified in two (22.2%) patients. Two (22.2%) patients were smokers. Diabetes was confirmed in two (22.2%) patients preoperatively. Two (22.2%) patients were hypertensive.

### Clinical Manifestations

The median interval between onset of symptoms and diagnosis of the duodenal polyp was 14 (range: 4–180) days. Five patients (55.5%) presented with clinical symptoms of upper GI hemorrhage either in the form of melena ( *n* = 5, 55.5%) or both hematemesis and melena ( *n* = 2, 22.2%). Three (33.3%) patients presented with abdominal pain and one (11.1%) patient presented with episodes of bilious vomiting.

### Investigations

Diagnostic endoscopy could detect the lesion in all (100%) the patients. Three (33.3%) of these lesions were pedunculated and rest six (66.6%) were sessile in nature. Contrast-enhanced computed tomography detected duodenal polypoidal lesion in five (55.5%) patients. Two (40%) of these five patients has extraluminal component of the lesion evidenced by loss of fat plane between duodenum and head of pancreas. EUS could characterize the submucosal lesions arising from duodenum accurately in all (100%) patients. Mean size of tumor was 4.78 ± 1.36 cm. Mean hemoglobin level was 8.6 ± 2.23 g/dL and mean serum albumin level was 4 ± 0.52 mg/dL.

### Treatment

Seven (77.7%) patients had undergone attempts of endoscopic removal before surgery. One patient had successful endoscopic removal 2 years ago but recurred. The type of surgery performed is presented in Table 1 . Open surgeries were done for all patients. Most commonly performed operation was duodenal polypectomy ( *n* = 6, 66.6%). The median operating time was 136 (range: 106–305) minutes. The median intraoperative blood loss was 150 (range: 100–240) mL. Six (66.6%) patients required blood transfusion. Median units of blood transfusion required was 2 (range: 1–4). Three postoperative complications developed in two (22.2%) patients. One (11.1%) patient developed wound infection and melena on third postoperative day. One patient developed type-B postoperative pancreatic fistula. Both the patients were managed conservatively. Median postoperative hospital stay was 8 (range: 6–11) days. There was no surgery-related mortality.

**Table 1:** Details of surgical procedures performed ( *n* = 9)

Procedures to remove polyp	*n* (%)
Duodenal polypectomy	6 (66.6)
Partial duodenectomy	1 (11.1)
Pancreaticoduodenectomy	2 (22.2)
Additional procedures	
Pyloroplasty	2 (22.2)

After a median follow-up of 60 (range: 12–78) months, no patient developed GI bleed or intestinal obstruction. Annual surveillance endoscopy was done which did not show any evidence of recurrence. One patient (11.1%) developed new onset DM following pancreaticodudenectomy.

## Discussion

Brunner's glands are alkaline mucin secreting glands located in the deep mucosal and submucosal layer of the duodenum. Functionally, it secretes alkaline fluid composed of mucin, pepsinogen, and enterogastrone which protect the duodenal mucosa from the injurious effect of gastric chyme. 7 These glands are abundant in the proximal duodenum and they decrease in number distally. 8 Feyrter classified the abnormal proliferation of Brunner's gland into the following three subtypes: (1) diffuse hyperplasia, (2) circumscribed hyperplasia, and (3) glandular adenoma. 9 Glandular adenoma is modified recently as BGH. Supporting evidences include the lack of encapsulation, absence of dysplasia, and the admixture of tissues including ducts, acini, smooth muscles, adipose tissue, lymphoid tissue, and smooth sheets of Brunner's gland, all contained within the pathological structure. 7 BGH is most commonly located in the posterior wall of the duodenal bulb. 10 Rarely, they may be found in the proximal jejunum. In our case series, they were found in the first ( *n* = 6, 66.6%) and second ( *n* = 3, 33.3%) part of the duodenum, respectively. These lesions generally presents as a single, sessile, or pedunculated polypoidal growths mostly in fifth and sixth decades of life with no sex predilection. 11 Clinical manifestations varies from asymptomatic lesion to GI hemorrhage. Majority of the patients develop anemia from chronic blood loss. Although some may present with melena or hematemesis. Melena is four times more common than hematemesis. 11 Similar results were found in our present study. In our study, one patient presented with massive upper GI bleed with hemodynamic instability. 2 Rare manifestations include obstructive jaundice, 12 pancreatitis, 13 and chronic diarrhea. 14 Provisional diagnosis of BGH is based on radiological, as well as endoscopic findings. In contrast studies, findings are nonspecific because there is usually a sessile or pedunculated filling defect in the duodenum, although it is useful in ruling out extraluminal extension of the lesion. Endoscopic biopsy findings are often noncontributory, as they cannot reach deep submucosal tumor tissue. 2 15 16 EUS characterizes the mass in terms of layer of origin, solid/cystic component, and vascularity. On EUS, BGH appears heterogeneous hypoechoic mass with multiple small cystic areas within the lesions and indistinct margins. Moreover, EUS is also helpful for obtaining fine needle aspiration from the submucosal lesion. It should be distinguished from other pathological differentials of duodenal polyp like leiomyoma, adenoma of the superficial mucosal glands, aberrant pancreatic tissue, and malignant tumors. 10 15 Regarding asymptomatic BGH found incidentally, whether it needs removal or not, is still a controversy. Although some authors have reported BGH causing acute profuse bleeding, resulting in shock. 11 17 Lesions larger than 2 cm are shown to cause more complications; hence, excision is recommended even if they are asymptomatic. Symptomatic lesions warrants excision. Endoscopic polypectomy is the first-line treatment for small or pedunculated lesions. Surgical excision is reserved for cases when tumor is too large or where snaring has failed as were the cases in our series. Moreover, doubtful lesions with extraluminal extension should be treated surgically. In our current study, most commonly performed operation was duodenal polypectomy ( *n* = 6, 66.6%). Our own unit does not have experience in advanced laparoscopic techniques, and hence we chose to remove the polyps at open procedures, but other centers might have approached the excision in other ways.

## Strengths and Limitations

There was no 90-day mortality and only one patient developed rebleeding in the perioperative period. Endoscopic surveillance is not recommended because of the very low malignant potential and rarity of recurrence after removal. 18 The study has some strength and limitations. The strength is that it is one of the largest surgical case series with no perioperative mortality and no newer studies have been performed in this area. The drawback is that it is a retrospective study, although prospective study for such a rare condition is not feasible.

## Conclusion

BGH should be considered in the differential diagnosis of all cases of polypoidal lesions of the duodenum. Reasonable outcomes can be obtained with expeditious management. Although, endoscopic management is the first-line treatment, surgery still plays an important role, particularly, if this fails or is not feasible. In experienced hand, surgery can be performed with acceptable perioperative morbidity and mortality.
